# Ultrasound Imaging Comparison of Crural Fascia Thickness and Muscle Stiffness in Stroke Patients with Spasticity

**DOI:** 10.3390/diagnostics14222606

**Published:** 2024-11-20

**Authors:** Jongwon Choi, Yerim Do, Haneul Lee

**Affiliations:** 1 Fascia Research Institute, Myofascial Release Korea, Seoul 06136, Republic of Korea; mfrkorea@gmail.com; 2 Department of Physical Therapy, The Graduate School, Gachon University, Incheon 21936, Republic of Korea; 3 Department and Research Institute of Rehabilitation Medicine, College of Medicine, Yonsei University, Seoul 03722, Republic of Korea; doyr62@gmail.com; 4 Department of Physical Therapy, College of Medical Science, Gachon University, Incheon 21936, Republic of Korea

**Keywords:** stroke, spasticity, fascia thickness, muscle stiffness, ultrasound imaging

## Abstract

Background/Objective: Spasticity following stroke causes structural changes in the muscles and fascia, affecting the mobility and functional recovery of patients. Understanding these structural changes is critical to optimizing the rehabilitation strategies for patients. Therefore, in this study, we aimed to investigate the differences in crural and epimysial fascia thickness and muscle stiffness in the affected and unaffected lower limbs of chronic stroke patients with spasticity. Methods: A total of 88 patients with chronic stroke (mean age: 62.7 ± 10.2 years) were included in this study. Ankle range of motion, crural fascial thickness, and muscle stiffness in affected and unaffected lower limbs were assessed using ultrasound. Results: For the affected lower limbs, 59 patients (67.1%) exhibited a modified Ashworth scale score of 2, whereas 29 patients (32.9%) exhibited a score of 3. Ankle range of motion, fascia thickness, and muscle stiffness were also measured. The range of motion in ankle dorsiflexion and plantar flexion was significantly reduced on the affected side (*p* < 0.05). Crural fascia thickness was significantly greater in all regions of the affected side (anterior: 0.96 ± 0.14 vs. 0.72 ± 0.08 mm [*p* < 0.001]; lateral: 1.01 ± 0.14 vs. 0.75 ± 0.14 mm [*p* < 0.001]), and the epimysial fascia of the tibialis anterior muscle was similarly greater in the affected side (0.46 ± 0.07 vs. 0.34 ± 0.03 mm [*p* < 0.001]). However, no significant differences in muscle stiffness were observed between the affected and unaffected sides (*p* > 0.05). Conclusions: Overall, these findings revealed significant fascial thickening with only minimal changes in muscle stiffness on the affected side, highlighting the importance of controlling fascial changes for post-stroke spasticity management.

## 1. Introduction

Approximately 12.8 million individuals worldwide experience a stroke annually, with spasticity observed in approximately 40% of all patients [1,2]. Spasticity, characterized by exaggerated tendon reflexes due to excessive activation of the stretch reflex, is commonly observed after neurological damage, such as stroke, traumatic brain injury, and spinal cord injury [3]. It occurs due to neural mechanisms causing excessive muscle activity, decreased relaxation, hypertonia, muscle spasms, and impaired motor control [4]. Reticulospinal hyperexcitability is a key cause of post-stroke spasticity, in which damage to the corticoreticular tract disinhibits the reticulospinal pathway, leading to excessive muscle tone [5]. Abnormal reorganization of motor circuits due to neural plasticity further exacerbates spasticity [6].

Fascia composed of dense and loose connective tissues plays key roles in muscle function and flexibility. Dense connective tissues provide strength and elasticity, whereas loose connective tissues, which are rich in hyaluronan, facilitate smooth gliding between the muscle and fascia [7]. However, without mechanical loading, the viscosity of hyaluronan increases, thereby hindering the gliding function of fascia [8]. Immobilization also increases the viscosity of hyaluronan, impeding fascial gliding and contributing to fascial thickening and fibrosis, particularly during paralysis [8,9]. This restricts gliding in the loose connective tissue layer of the fascia, thereby causing muscle stiffness [10]. Increased hyaluronan levels also increase the fascial viscoelasticity, alter the muscle spindle sensitivity, and exacerbate spasticity [9,11].

Fascia is a crucial tissue interacting with the musculoskeletal and nervous systems [12]. It is closely linked to both muscles and nerves; therefore, changes in the nervous system directly affect the fascial structure and function. Post-brain-injury changes in neurotransmitter levels cause pathological changes in the fascia [13]. Increases in inflammatory cytokine levels and changes in neurotrophic factors (nerve growth and brain-derived neurotrophic factors) after brain injury cause fascial fibrosis, leading to decreased muscle flexibility and function. Activation of inflammatory responses increases the fascia thickness, hindering nerve and muscle regeneration [14]. These changes reduce the fascial flexibility, limit muscle movement, and negatively affect the functional recovery of patients. Lower limb muscles and fascia play critical roles in the mobility and daily functions of patients with stroke [12]. As lower limb muscles are involved in essential movements such as walking, fascial-fibrosis-induced stiffness and reduced flexibility severely impair the motor ability and rehabilitation of patients [14,15]. Fascial fibrosis results in chronic pain and movement restriction, thereby adversely affecting patient rehabilitation [7]. Increased fascia thickness further limits the joint range of motion (ROM), resulting in chronic pain and impairing the rehabilitation of stroke patients [7].

Owing to its high resolution and non-invasive nature, ultrasound imaging is widely used to assess soft tissue structure in clinical practice [16]. It accurately detects increased hyaluronan density manifesting as fascial thickening [7]. Shear wave elastography (SWE) is an advanced ultrasound technique for quantifying tissue stiffness, providing quantitative elastic information in both Young’s modulus, measured in kPa, and shear wave velocity, measured in m/s [17,18,19]. There is a direct relationship between Young’s modulus and shear wave propagation speed, explained by the formula *E* = 3*ρ*c^2^ (where *E* represents Young’s modulus, *ρ* is tissue density in kg/m^3^, and c is the shear wave propagation velocity) [19], based on the assumption of isotropic and homogeneous media [20].

Previous studies using SWE have reported significantly greater shear elastic modulus in the affected muscles of patients with stroke than in the muscles of healthy individuals, indicating abnormal post-stroke muscle changes [21]. Although previous studies have advanced our understanding of fascial changes and muscle stiffness in stroke patients, no study has specifically investigated the differences in fascia thickness of the affected and unaffected sides to date. Therefore, this cross-sectional study aimed to compare the crural and epimysial fascia thickness and muscle stiffness of the affected and unaffected sides in chronic stroke patients with spasticity.

## 2. Materials and Methods

### 2.1. Ethical Consideration

This observational study was performed according to the Strengthening the Reporting of Observational Studies in Epidemiology guidelines [22] and adhered to the Declaration of Helsinki. This study was approved by the Gachon University Institutional Review Board (1044396-202403-HR-029-01). All participants were informed of the study purpose and experimental procedures and signed informed consent forms prior to their inclusion in the study.

### 2.2. Participants

In total, 100 patients with chronic stroke were initially included in this study. Among these, 4 did not meet the inclusion criteria and 8 refused to participate in the study; therefore, 88 patients were finally evaluated in the study. The inclusion criteria were as follows: diagnosis of hemiplegia due to stroke six months after onset and a modified Ashworth scale (MAS) spasticity grade ≥ 2 in the lower limbs. The exclusion criteria were as follows: presence of cognitive impairments or dementia with a Korean version of the mini-mental state examination score ≤ 18, inability to understand instructions, and diagnosis of musculoskeletal injuries or conditions causing lower limb pain within the last six months. Patients with body mass index ≥ 30 kg/m^2^ were also excluded from the study, as a high body mass index increases the subcutaneous fat layer, which enhances the reflection and absorption of ultrasound signals, thereby reducing the measurement accuracy [23].

Sample size was determined using the G-power 3.1.9 software (Heinrich Heine University Dusseldorf, Dusseldorf, Germany). To calculate the sample size, probability of an alpha error and power were set at 0.05 and 0.8, respectively, with a small effect size (d = 0.3) based on Cohen’s report [24]. A sample size of 90 participants was necessary to ensure statistical validity.

### 2.3. Measurements

#### 2.3.1. Passive Ankle Joint ROM

Ankle joint ROM was measured using a goniometer, with the participants positioned supine, knees extended, and ankles hanging off the edge of the bed. Dorsiflexion was measured from maximum plantar flexion to maximum dorsiflexion, and plantar flexion was measured from maximum dorsiflexion to maximum plantar flexion. ROM from maximum plantar flexion to dorsiflexion was 70°. The goniometer axis was aligned with the lateral malleolus, the stationary arm along the lateral midline of fibula, and the moving arm along the lateral midline of the fifth metatarsal. The investigator passively moved the ankle to the end of ROM until resistance was felt. This position was used to accommodate patients with stroke who faced discomfort in the prone or seated positions, and measurements were taken at the most comfortable knee angle for each patient (0–30° of flexion).

#### 2.3.2. Fascia Thickness

Crural and epimysial fascia thickness values were measured using ultrasonography (RS85 Prestige, Samsung Medicine, Seoul, Republic of Korea) using a 2–14 MHz linear transducer. The crural fascia is the deep fascia of the leg that surrounds it tightly and connects superiorly with the fascia lata of the thigh, attaching around the knee and to the tibia and fibula [25]. The epimysial fascia, or epimysium, is a connective tissue sheath surrounding skeletal muscles, often connecting to the periosteum of bones and tightly binding to the muscle through septa [26]. Measurements were taken for the anterior, lateral, and posterior compartments of the crural fascia using specific anatomical landmarks, as previously described [27]. However, the patient position was adjusted according to the specific characteristics of the patients with stroke to ensure accurate and comfortable measurements. For crural fascia, measurements were taken at three points in the anterior compartment and two points in the lateral compartment, and average values were used for analysis of each compartment. As the posterior compartment is divided into three distinct muscles (medial and lateral gastrocnemius and soleus muscles), the three points were analyzed separately. Considering the difficulty in distinguishing epimysial fascia from crural fascia in calves using ultrasound, measurements of epimysial fascia were only taken at the tibialis anterior muscle, which is easy to differentiate [27]. A probe was applied to the skin with minimal pressure to prevent tissue compression and ensure stable contact for consistent imaging. To minimize the effects of thickness variations, measurements were taken in three evenly spaced regions of interest in each muscle layer.

**Figure 1:**
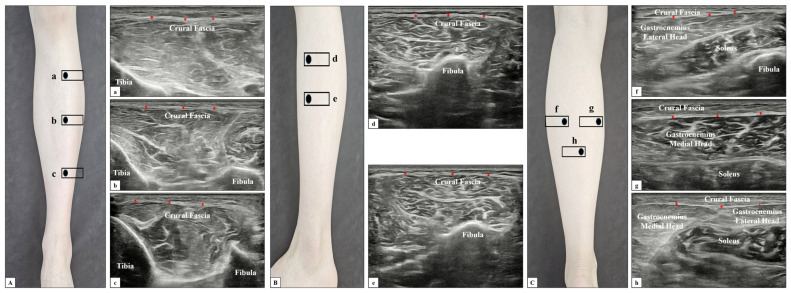
Ultrasound images of crural fascia thickness in the anterior (**A**), lateral (**B**), and posterior (**C**) compartments. (**A**) Probe at three positions in the anterior compartment (**a**–**c**), with corresponding ultrasound images indicating crural fascia measurements 1–3. (**B**) Probe at two positions in the lateral compartment (**d**,**e**), with corresponding ultrasound images indicating crural fascia measurements 1–2. (**C**) Probe at three positions in the posterior compartment (**f**–**h**), with corresponding ultrasound images indicating crural fascia measurements 1–3. Red arrows indicate the thickness of crural fascia.

Anterior compartment: Patients were positioned supine with the calf in a neutral position. Measurements were taken at three locations (Figure 1A): (a) proximal third of the anterior calf below the tibial crest, (b) middle third of the anterior calf over the belly of the tibialis anterior muscle, and (c) distal third of the anterior calf above the flexor retinaculum.

Lateral compartment: Patients were positioned supine with the calf in a neutral position. Measurements were taken at two locations (Figure 1B): (d) proximal third of the lateral calf over the head of the fibula and peroneus longus muscle, and (e) distal to and (d) along the fibula, where the peroneus longus and brevis muscles overlapped.

Posterior compartment: Patients were positioned supine on an adjustable table with the calf hanging off the edge due to spasticity and contracture in the arms of patients with stroke. Measurements were taken at three locations (Figure 1C): (f) over the medial gastrocnemius muscle belly, (g) over the lateral gastrocnemius muscle belly, and (h) following the sural nerve and veins downward to the point of convergence of the gastrocnemius and soleus muscles.

**Figure 2:**
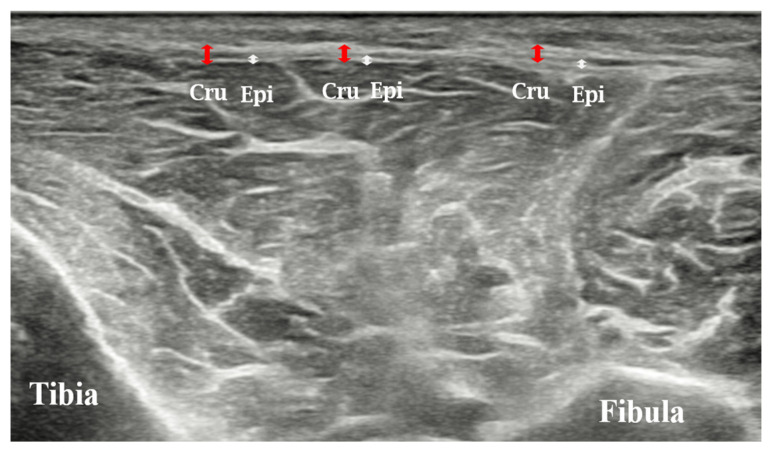
Ultrasound image of the thickness values of crural fascia and epimysial fascia of the tibialis anterior muscle. Red arrows indicate the thickness of crural fascia, and white arrows indicate the thickness of the epimysial fascia. Abbreviations: Cru, crural fascia; Epi, epimysial fascia.

Epimysial fascia of the tibialis anterior muscle: Patients were positioned supine with the calf in a neutral position. Measurements were only taken at the point (Figure 1b) where the epimysial fascia of the tibialis anterior muscle could be distinctly identified below the crural fascia using ultrasound (Figure 2).

Previous research confirmed good intra-rater reliability for the ultrasound device in measuring crural and epimysial fascia thickness, with an ICC of 0.83 (95% CI: 0.73–0.91) for crural fascia and 0.82 (95% CI: 0.67–0.91) for epimysial fascia [27]. 

#### 2.3.3. Muscle Stiffness

**Figure 3:**
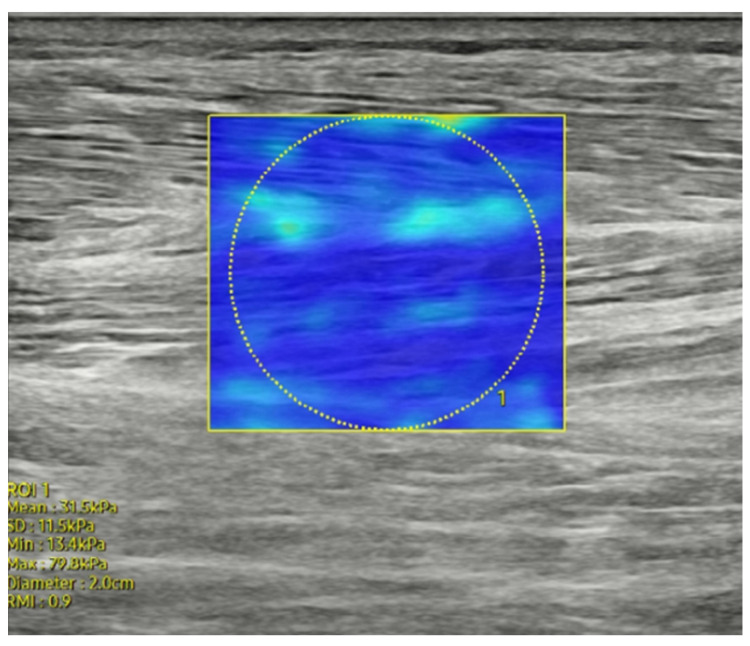
Shear wave elastography image of the tibialis anterior muscle stiffness.

RS85 Prestige ultrasound unit coupled to a 2–14 MHz linear probe (RS85 Prestige, Samsung Medicine, Republic of Korea) was used to assess the muscle stiffness in lower limbs, with Young’s modulus measured in kPa, which serves as a proxy for stiffness in biological tissues. The transducer was aligned longitudinally in the direction of the muscles following standard practice to ensure accurate stiffness measurements. The measurements were conducted by a physical therapist with over 10 years of experience specializing in fascia-focused treatments and extensive practice in cadaveric dissection of fascia, as well as expertise in using ultrasound technology. Measurements were taken over the muscle bellies of the tibialis anterior (anterior compartment), peroneus longus (lateral compartment), medial and lateral gastrocnemius, and soleus (posterior compartment) muscles in the resting position, identical to the positions used for fascia thickness measurement. Software-generated color map and quantitative stiffness values were obtained using an acoustic push pulse followed by a detection pulse. In SWE images, red regions represent areas with high shear wave velocity, while blue regions indicate lower shear wave velocity, serving as proxies for tissue stiffness. The region of interest, defined as a circular area (2.2 cm), was centrally positioned over the target muscle (Figure 3). Stiffness is expressed as kPa or m/s, depending on the depth. Both mean and median values were calculated. In this manuscript, muscle stiffness is specifically expressed as kPa. 

### 2.4. Statistical Analysis

Data were analyzed using SPSS for Windows (version 22.0; IBM Corp., Armonk, NY, USA). Data were calculated as the means and standard deviations for quantitative variables and frequencies and percentages for categorical variables. The Shapiro–Wilk test was used to assess the data normality. A repeated-measures ANOVA was used to compare differences in ankle joint ROM, thickness of the crural and epimysial fascia, and muscle stiffness between the affected and unaffected sides, considering within-subject variability. To control for multiple comparisons, confidence interval adjustment for the estimated marginal means was performed using the Bonferroni correction. Statistical significance was set at α = 0.05.

## 3. Results

In this study, the average age of the participants was 62.7 years, with 36.4% being female. The average number of months since diagnosis was 56.6 months. The left side was affected in 51 (58%) participants. Hemiplegia was due to infarction in 46 participants (52.3%) and hemorrhage in 42 participants (47.7%). The MAS score of spasticity in the lower limb was 2 in 59 participants (67.1%) and 3 in 29 participants (32.9%; Table 1).

### 3.1. Comparision of Ankle ROM in the Affected and Unaffected Sides

The analysis revealed a significant main effect of side on ankle ROM, F_(1,87)_ = 205.97, *p* < 0.001, partial η^2^ = 0.703, indicating that ROM differed significantly between the affected and unaffected sides. Additionally, a significant main effect was observed for the ROM (dorsiflexion vs. plantarflexion), F_(1,87)_ = 239.34, *p* < 0.001, partial η^2^ = 0.733, suggesting significant range between dorsiflexion and plantarflexion. The interaction effect between side and ROM type was also significant, F_(1,87)_ = 90.50, *p* < 0.001, partial η^2^ = 0.501, indicating that the difference in ROM between the affected and unaffected sides varied depending on the movement type.

Pairwise comparisons with Bonferroni adjustment further confirmed that dorsiflexion ROM was significantly reduced on the affected side compared to the unaffected side (40.11 ± 12.51° vs. 58.14 ± 7.71°, *p* < 0.001). Similarly, plantarflexion ROM was slightly lower on the affected side than on the unaffected side (65.67 ± 7.01° vs. 67.38 ± 4.80°, *p* < 0.05).

### 3.2. Comparision Comparison of Crural and Epimysial Fascia Thickness on the Affected and Unaffected Sides

The analysis revealed a significant main effect of side on fascia thickness, F_(1,87)_ = 465.01, *p* < 0.001, partial η^2^ = 0.842, indicating that the affected side exhibited significantly thicker fascia than the unaffected side across all measured regions. Additionally, a significant main effect was observed for the specific fascia regions, F_(5,83)_ = 232.57, *p* < 0.001, partial η^2^ = 0.728, highlighting distinct thicknesses among the various fascia regions. There was also a significant interaction effect between side and fascia region, F_(5,83)_ = 5.51, *p* < 0.001, partial η^2^ = 0.060, suggesting that the difference in thickness between the affected and unaffected sides varied across specific fascia regions.

Pairwise comparisons with Bonferroni adjustment further confirmed that each fascia region showed greater thickness on the affected side compared to the unaffected side (all *p* < 0.001; Table 2). In the anterior region, crural fascia thickness was 0.96 ± 0.14 mm on the affected side and 0.72 ± 0.08 mm on the unaffected side. Similarly, in the lateral compartment, crural fascia thickness was 1.01 ± 0.14 mm on the affected side and 0.75 ± 0.14 mm on the unaffected side. In the posterior compartments, significant differences were observed as follows: crural fascia thickness was 0.92 ± 0.22 mm versus 0.68 ± 0.11 mm (affected vs. unaffected side) in posterior region 1, 0.96 ± 0.21 mm versus 0.72 ± 0.16 mm in posterior region 2, and 1.02 ± 0.30 mm versus 0.77 ± 0.16 mm in posterior region 3. Additionally, thickness of the epimysial fascia of the tibialis anterior muscle also differed significantly, measuring 0.46 ± 0.07 mm on the affected side and 0.34 ± 0.03 mm on the unaffected side.

### 3.3. Comparision of Lower Limb Muscle Stiffness on the Affected and Unaffected Sides

The analysis revealed no significant main effect of side on muscle stiffness, F_(1,87)_ = 0.12, *p* = 0.737, indicating that the affected side showed no significant difference across all measured regions. Additionally, a significant main effect was observed for the specific muscle regions, F_(5,83)_ = 70.07, *p* < 0.001, partial η^2^ = 0.599, showing significant muscle stiffness among different muscles. There was also no significant interaction effect between side and muscle stiffness, F_(5,83)_ = 1.14, *p* = 0.339.

Pairwise comparisons with Bonferroni adjustment further confirmed that all muscle stiffness values did not show differences in the stiffness between the affected and unaffected sides except for the medial gastrocnemius muscle (24.10 ± 5.06 vs. 22.26 ± 4.91 kPa, *p* = 0.009; Table 3).

## 4. Discussion

This cross-sectional study revealed significant differences in crural and epimysial fascia thickness between the affected and unaffected sides of chronic stroke patients with spasticity. The results showed that the fascia thickness was approximately 30% greater on the affected side in all regions of the crural fascia. In contrast, muscle stiffness was not significantly different between the affected and unaffected sides for most muscles.

Fascia consists of dense and loose connective tissue layers. The dense layer provides strength and elasticity via collagen and elastin fibers, whereas the loose layer, which is rich in extracellular matrix, aids in shock absorption and immune responses [28]. Under conditions such as spasticity and immobility, prolonged inactivity leads to fibrosis and increased collagen deposition in the dense layer and hyaluronan accumulation in the loose layer, thereby contributing to fascial thickening [7]. These structural changes may be responsible for the increased fascia thickness observed in this study. Consistently, Foran et al. reported increased collagen deposition and fibrosis in the fascial tissue of spastic muscles in patients with neurological impairments [29]. Similarly, spasticity is linked to an increase in extracellular matrix, which contributes to both muscle contracture and stiffness in patients with cerebral palsy [30]. Hyaluronan accumulation in the fascia, as noted by Stecco et al., leads to reduced mobility and flexibility [9]. This reduced mobility further contributes to structural changes, such as increased fascia thickness in the affected side in patients with stroke, which can subsequently impact functional recovery by restricting the sliding of muscle compartments. Hyaluronan, abundant in the endomysium, perimysium, and epimysium, normally facilitates muscle sliding through lubrication [31]. However, prolonged immobility increases hyaluronan concentration and fluid viscosity within these fascial layers, potentially leading to decreased sliding between collagen fibers and a patient’s perception of stiffness [8,10]. These findings are consistent with those of our study, which showed that the affected side has a thicker fascia than the unaffected side in patients with stroke.

Regarding the muscle stiffness in the lower limbs, no significant differences were observed between the affected and unaffected sides, except for the medial gastrocnemius muscles, contradicting previous reports on muscle stiffness in the spastic muscles of patients with stroke and healthy individuals. For example, Eby et al. reported greater muscle stiffness on the affected side in stroke patients than in healthy individuals [32]. However, other studies reported no significant differences in muscle stiffness between the affected side of patients and healthy controls under resting conditions [33,34].

In this study, the observed differences in fascia thickness and muscle stiffness were possibly due to their distinct roles in spasticity. This study demonstrated significant differences in fascia thickness on the affected and unaffected sides; however, no such differences were observed in the stiffness of most muscles. Fascial thickening, as observed in this study, is possibly a long-term structural change resulting from prolonged immobility and fibrosis of the connective tissue. While SWE is valuable for quantifying tissue stiffness, its application in biological tissues like muscle presents specific limitations. Muscle tissues are inherently anisotropic and heterogeneous, meaning that stiffness measurements can vary with the orientation of the ultrasound transducer relative to the muscle fiber direction [21]. This variability may introduce inconsistencies in Young’s modulus measurements, especially in tissues with complex fiber orientations such as muscle. Recognizing these limitations allows for a nuanced interpretation of our results, especially in clinical contexts where measurement accuracy is critical [21]. Despite these limitations, studies have demonstrated SWE’s utility in quantifying elastic properties across various healthy and pathological connective tissues, including tendons and muscles [35,36,37].

Ultrasound measurements have shown that increased collagen deposition and hyaluronan viscosity in the fascia leads to reduced flexibility and increased stiffness [7,38]. In contrast, muscle stiffness, particularly during rest, is influenced more by neural factors, such as the degree of spasticity or overactivity of stretch reflexes, rather than by structural changes in the muscle tissues [9,39]. Spasticity is primarily caused by neural overactivity, which is minimized during resting [40]; therefore, muscle stiffness differences were not significant in most muscles during rest in this study. Subtle changes in the unaffected side due to post-stroke compensatory mechanisms reduce the differences in muscle stiffness between both sides [41,42]. To address the fascial changes identified in this study, targeted therapeutic interventions may help improve function by reducing fascial stiffness. For example, fascial manipulation techniques have been effective in improving ROM and reducing pain [9]. Additionally, hyaluronidase injections in patients with spasticity have shown the potential to increase joint movement and reduce stiffness by lowering hyaluronan viscosity in the fascia [43].

While fascial thickening may contribute to spasticity by increasing mechanical resistance and restricting movement, it could also be a secondary change resulting from prolonged spasticity and reduced mobility rather than an immediate consequence of CNS injury [44]. It is plausible that structural changes in and around skeletal muscles, including fascial thickening, may contribute to the development of spasticity following CNS injury [9]. Importantly, these changes do not necessarily correlate with increased muscle stiffness under resting conditions, highlighting the distinct contributions of neural and structural factors in spasticity. To address these structural changes, targeted therapeutic interventions such as fascial manipulation may provide clinical benefits by reducing fascial stiffness and enhancing mobility.

The increase in fascial thickness observed in our study aligns with findings from previous research, which also identified fascial thickening in athletes with unstable ankles. This reported increased epimysial fascia thickness in athletes with unstable ankles, suggesting that structural changes in the fascia may occur post-injury [27]. The consistency of our findings supports the applicability of fascial thickness measurements to other patient populations, emphasizing the importance of fascial assessment not only in stroke patients but also in various clinical settings where spasticity and restricted movement are present. It demonstrates that ultrasound-based measurements of fascial thickness are reliable and clinically meaningful across different groups.

To the best of our knowledge, this study is the first to compare the fascia thickness and muscle stiffness between the affected and unaffected sides of chronic stroke patients with spasticity; however, it has several limitations. First, the wide age range of participants (24–89 years old) possibly affected the measurement consistency as age influences body composition and tissue properties [45]. Second, variation in stroke onset time from six months to over 10 years (185 months) may have introduced variability in the results, with acute and chronic tissue changes affecting the results differently over time [46]. These factors may have influenced the observed differences in muscle stiffness and fascia thickness in this study. Additionally, regarding SWE, although prior studies have demonstrated good reliability in ultrasound measurements of fascia thickness [27], this study did not directly assess the reliability of our measurements, which may have affected consistency. Finally, the final sample size of 88 participants was slightly below the target of 90 participants indicated by the power analysis. Although this may have marginally reduced the statistical power, it did not significantly affect the overall findings of this study.

## 5. Conclusions

This study demonstrated the significant differences in crural and epimysial fascia thickness, with no differences in stiffness, between the affected and unaffected sides in chronic stroke patients with spasticity. Our findings suggest that post-stroke fascial thickening may contribute to restricted movement, potentially playing a key role in limiting joint mobility and contributing to functional impairment. Clinically, these findings highlight the need for therapeutic approaches that specifically target fascial thickening rather than solely focusing on muscle stiffness. Future studies should further investigate the role of fascial changes during rehabilitation to optimize strategies for improving mobility and reducing spasticity-related impairments in stroke patients.

## Figures and Tables

**Table 1:** General characteristics of the participants (*N* = 88).

Characteristics	*N* (%)	Mean ± SD
Age (years)		62.7 ± 13.0
Sex (female)	32 (36.4)	
Affected side (right)	37 (42.0)	
BMI (kg/m^2^)		23.4 ± 2.9
Stroke type		
Infarction	46 (52.3)	
Hemorrhage	42 (47.7)	
Onset (months)		56.6 ± 45.3
MAS		
2	59 (67.1)	
3	29 (32.9)	

Abbreviations: BMI, body mass index; MAS, modified Ashworth scale.

**Table 2:** Measurement of crural and epimysial fascia thickness on the affected and unaffected sides (*N* = 88).

Characteristics	Affected Side	Unaffected Side	*p*
Anterior compartment of crural fascia	0.96 ± 0.14	0.72 ± 0.08	<0.001
Lateral compartment of crural fascia	1.01 ± 0.14	0.75 ± 0.14	<0.001
Posterior compartment of crural fascia 1	0.92 ± 0.22	0.68 ± 0.11	<0.001
Posterior compartment of crural fascia 2	0.96 ± 0.21	0.72 ± 0.16	<0.001
Posterior compartment of crural fascia 3	1.02 ± 0.30	0.77 ± 0.16	<0.001
Tibialis anterior epimysial fascia	0.46 ± 0.07	0.34 ± 0.03	<0.001

**Table 3:** Measurement of muscle stiffness on the affected and unaffected sides (*N* = 88).

Characteristics	Affected Side	Unaffected Side	*p*
Tibialis anterior	27.32 ± 6.07	27.40 ± 6.23	0.931
Peroneus longus	22.01 ± 4.67	21.99 ± 4.57	0.980
Medial gastrocnemius	24.10 ± 5.06	22.26 ± 4.91	0.009
Lateral gastrocnemius	24.31 ± 5.71	24.09 ± 6.29	0.825
Soleus	37.56 ± 11.14	36.49 ± 10.75	0.507

## Data Availability

The datasets generated in this study are available from the corresponding author upon request.

## References

[1] Feigin V.L., Brainin M., Norrving B., Martins S., Sacco R.L., Hacke W., Fisher M., Pandian J., Lindsay P. (2022). World Stroke Organization (WSO): Global Stroke Fact Sheet 2022. Int. J. Stroke.

[2] Glaess-Leistner S., Ri S.J., Audebert H.J., Wissel J. (2021). Early clinical predictors of post stroke spasticity. Top. Stroke Rehabil..

[3] Nair K.P., Marsden J. (2014). The management of spasticity in adults. BMJ.

[4] Kheder A., Nair K.P. (2012). Spasticity: Pathophysiology, evaluation and management. Pract. Neurol..

[5] Mahmoud W., Hultborn H., Zuluaga J., Zrenner C., Zrenner B., Ziemann U., Ramos-Murguialday A. (2023). Testing spasticity mechanisms in chronic stroke before and after intervention with contralesional motor cortex 1 Hz rTMS and physiotherapy. J. NeuroEngineering Rehabil..

[6] Li S., Francisco G.E. (2015). New insights into the pathophysiology of post-stroke spasticity. Front. Hum. Neurosci..

[7] Pavan P.G., Stecco A., Stern R., Stecco C. (2014). Painful connections: Densification versus fibrosis of fascia. Curr. Pain. Headache Rep..

[8] Cowman M.K., Schmidt T.A., Raghavan P., Stecco A. (2015). Viscoelastic Properties of Hyaluronan in Physiological Conditions. F1000Research.

[9] Stecco A., Stecco C., Raghavan P. (2014). Peripheral Mechanisms Contributing to Spasticity and Implications for Treatment. Curr. Phys. Med. Rehabil. Rep..

[10] Stecco A., Gesi M., Stecco C., Stern R. (2013). Fascial components of the myofascial pain syndrome. Curr. Pain. Headache Rep..

[11] Matteini P., Dei L., Carretti E., Volpi N., Goti A., Pini R. (2009). Structural behavior of highly concentrated hyaluronan. Biomacromolecules.

[12] Langevin H.M. (2021). Fascia Mobility, Proprioception, and Myofascial Pain. Life.

[13] Dorsett C.R., McGuire J.L., DePasquale E.A., Gardner A.E., Floyd C.L., McCullumsmith R.E. (2017). Glutamate Neurotransmission in Rodent Models of Traumatic Brain Injury. J. Neurotrauma.

[14] Kodama Y., Masuda S., Ohmori T., Kanamaru A., Tanaka M., Sakaguchi T., Nakagawa M. (2023). Response to Mechanical Properties and Physiological Challenges of Fascia: Diagnosis and Rehabilitative Therapeutic Intervention for Myofascial System Disorders. Bioengineering.

[15] Zullo A., Fleckenstein J., Schleip R., Hoppe K., Wearing S., Klingler W. (2020). Structural and Functional Changes in the Coupling of Fascial Tissue, Skeletal Muscle, and Nerves During Aging. Front. Physiol..

[16] Forney M.C., Delzell P.B. (2018). Musculoskeletal ultrasonography basics. Cleve Clin. J. Med..

[17] Taljanovic M.S., Gimber L.H., Becker G.W., Latt L.D., Klauser A.S., Melville D.M., Gao L., Witte R.S. (2017). Shear-Wave Elastography: Basic Physics and Musculoskeletal Applications. Radiographics.

[18] Lim Y., Do Y., Lee H. (2024). Association between abdominal muscle stiffness, diaphragm thickness and peak expiratory flow in younger versus older adults. Clin. Physiol. Funct. Imaging.

[19] Youk J.H., Son E.J., Park A.Y., Kim J.A. (2014). Shear-wave elastography for breast masses: Local shear wave speed (m/sec) versus Young modulus (kPa). Ultrasonography.

[20] Ryu J., Jeong W.K. (2017). Current status of musculoskeletal application of shear wave elastography. Ultrasonography.

[21] Leng Y., Wang Z., Bian R., Lo W.L.A., Xie X., Wang R., Huang D., Li L. (2019). Alterations of Elastic Property of Spastic Muscle With Its Joint Resistance Evaluated From Shear Wave Elastography and Biomechanical Model. Front. Neurol..

[22] von Elm E., Altman D.G., Egger M., Pocock S.J., Gøtzsche P.C., Vandenbroucke J.P. (2007). The Strengthening the Reporting of Observational Studies in Epidemiology (STROBE) statement: Guidelines for reporting observational studies. Lancet.

[23] Kharaji G., ShahAli S., Ebrahimi Takamjani I., Kashanian M., Sarrafzadeh J., Shanbehzadeh S. (2023). Ultrasound assessment of the abdominal, diaphragm, and pelvic floor muscles during the respiratory and postural tasks in women with and without postpartum lumbopelvic pain: A case-control study. Int. Urogynecol. J..

[24] Cohen J. (2013). Statistical Power Analysis for the Behavioral Sciences.

[25] Wineski L.E. (2024). Snell’s Clinical Anatomy by Regions.

[26] Gatt A., Agarwal S., Zito P.M. (2024). Anatomy, Fascia Layers. StatPearls.

[27] Pirri C., Fede C., Stecco A., Guidolin D., Fan C., De Caro R., Stecco C. (2021). Ultrasound Imaging of Crural Fascia and Epimysial Fascia Thicknesses in Basketball Players with Previous Ankle Sprains Versus Healthy Subjects. Diagnostics.

[28] Stecco C., Gagey O., Macchi V., Porzionato A., De Caro R., Aldegheri R., Delmas V. (2007). Tendinous muscular insertions onto the deep fascia of the upper limb. First part: Anatomical study. Morphologie.

[29] Foran J.R., Steinman S., Barash I., Chambers H.G., Lieber R.L. (2005). Structural and mechanical alterations in spastic skeletal muscle. Dev. Med. Child. Neurol..

[30] Smith L.R., Lee K.S., Ward S.R., Chambers H.G., Lieber R.L. (2011). Hamstring contractures in children with spastic cerebral palsy result from a stiffer extracellular matrix and increased in vivo sarcomere length. J. Physiol..

[31] Huijing P.A., Jaspers R.T. (2005). Adaptation of muscle size and myofascial force transmission: A review and some new experimental results. Scand. J. Med. Sci. Sports.

[32] Eby S., Zhao H., Song P., Vareberg B.J., Kinnick R., Greenleaf J.F., An K.N., Chen S., Brown A.W. (2016). Quantitative Evaluation of Passive Muscle Stiffness in Chronic Stroke. Am. J. Phys. Med. Rehabil..

[33] Wu C.H., Ho Y.C., Hsiao M.Y., Chen W.S., Wang T.G. (2017). Evaluation of Post-Stroke Spastic Muscle Stiffness Using Shear Wave Ultrasound Elastography. Ultrasound Med. Biol..

[34] Mathevon L., Michel F., Aubry S., Testa R., Lapole T., Boulard C., Fernandez B., Parratte B., Calmels P. (2015). Reliability of 2D ultrasound imaging associated with transient ShearWave Elastography method to analyze spastic gastrocnemius medialis muscle architecture and viscoelastic properties. Ann. Phys. Rehabil. Med..

[35] Feng Y.N., Li Y.P., Liu C.L., Zhang Z.J. (2018). Assessing the elastic properties of skeletal muscle and tendon using shearwave ultrasound elastography and MyotonPRO. Sci. Rep..

[36] Do Y., Lall P.S., Lee H. (2021). Assessing the Effects of Aging on Muscle Stiffness Using Shear Wave Elastography and Myotonometer. Healthcare.

[37] Lee Y., Kim M., Lee H. (2021). The Measurement of Stiffness for Major Muscles with Shear Wave Elastography and Myoton: A Quantitative Analysis Study. Diagnostics.

[38] Pratt R.L. (2021). Hyaluronan and the Fascial Frontier. Int. J. Mol. Sci..

[39] Raghavan P., Raghavan P. (2022). Framework for the Treatment of Spasticity and Muscle Stiffness. Spasticity and Muscle Stiffness: Restoring Form and Function.

[40] Wang H., Huang P., Li X., Samuel O.W., Xiang Y., Li G. (2019). Spasticity Assessment Based on the Maximum Isometrics Voluntary Contraction of Upper Limb Muscles in Post-stroke Hemiplegia. Front. Neurol..

[41] Jones T.A. (2017). Motor compensation and its effects on neural reorganization after stroke. Nat. Rev. Neurosci..

[42] Brancaccio A., Tabarelli D., Belardinelli P. (2022). A New Framework to Interpret Individual Inter-Hemispheric Compensatory Communication after Stroke. J. Pers. Med..

[43] Raghavan P., Lu Y., Mirchandani M., Stecco A. (2016). Human Recombinant Hyaluronidase Injections For Upper Limb Muscle Stiffness in Individuals With Cerebral Injury: A Case Series. EBioMedicine.

[44] Watkins C.L., Leathley M.J., Gregson J.M., Moore A.P., Smith T.L., Sharma A.K. (2002). Prevalence of spasticity post stroke. Clin. Rehabil..

[45] Wilke J., Macchi V., De Caro R., Stecco C. (2019). Fascia thickness, aging and flexibility: Is there an association?. J. Anat..

[46] Azzollini V., Dalise S., Chisari C. (2021). How Does Stroke Affect Skeletal Muscle? State of the Art and Rehabilitation Perspective. Front. Neurol..

